# Mitochondrial genomes provide insights into the Euholognatha (Insecta: Plecoptera)

**DOI:** 10.1186/s12862-024-02205-6

**Published:** 2024-02-01

**Authors:** Jin-Jun Cao, Ying Wang, Dávid Murányi, Jian-Xin Cui, Wei-Hai Li

**Affiliations:** 1 Henan International Joint Laboratory of Taxonomy and Systematic Evolution of Insecta, Henan Institute of Science and Technology, Henan, 453003 China; 2 Department of Zoology, Eszterházy Károly Catholic University, Leányka u. 6, Eger, H-3300 Hungary

**Keywords:** Stonefly, Mitochondrial genome, Compositional heterogeneity, Phylogeny

## Abstract

**Background:**

Euholognatha is a monophyletic group within stoneflies comprised by a superfamily Nemouroidea and a family Scopuridae. Based on morphological data, the family-level phylogenetic relationships within Euholognatha are widely accepted, but there is still controversy among different molecular studies. To better understand the phylogeny of all six extant euholognathan families, we sequenced and analyzed seven euholognathan mitogenomes.

**Results:**

The sequence heterogeneity analysis observed a low degree of compositional heterogeneity in euholognathan mitogenomes. Meanwhile, leuctrid mitogenomes were more heterogeneous than other euholognathan families, which may affect the phylogenetic reconstruction. Phylogenetic analyses with various datasets generated three topologies. The Leuctridae was recovered as the earliest branching lineage, and the sister relationship of Capniidae and Taeniopterygidae was supported by most tree topologies and FcLM analyses. When separately excluding sparsely sampled Scopuridae or high heterogeneity leuctrid taxa, phylogenetic analyses under the same methods generated more stable and consistent tree topologies. Finally, based on the results of this study, we reconstructed the relationships within Euholognatha as: Leuctridae + (Scopuridae + ((Taeniopterygidae + Capniidae) + (Nemouridae + Notonemouridae))).

**Conclusion:**

Our research shows the potential of data optimizing strategies in reconstructing phylogeny within Euholognatha and provides new insight into the phylogeny of this group.

**Supplementary Information:**

The online version contains supplementary material available at 10.1186/s12862-024-02205-6.

## Introduction

The Plecoptera (also called stoneflies) is a group of hemimetabolous aquatic insects that includes over 4,400 extant species [1, 2]. Stonefly nymphs are important members of the stream ecosystem. They are frequently employed as bioindicators for monitoring the quality of water because their nymphs are extremely sensitive to water quality [3]. In addition, some nymphs can be used as the diet of fish and invertebrate predators [4]. Due to their important ecological and economic value, the taxonomy and systematics of stoneflies have become a research hotspot nowadays.

In the last few decades, the higher classification of Plecoptera has undergone numerous revisions [5–9]. In 2000, Zwick revised the higher classification of the Plecoptera and proposed a widely accepted classification system for stoneflies [1]. The infraorder Euholognatha, which belongs to the suborder Arctoperlaria, was recovered as a monophyletic group in Zwick’s study [1]. The euholognathan species are mainly distributed in the Northern Hemisphere, comprising a superfamily (Nemouroidea) and a family (Scopuridae) [1, 2]. The former includes five families: Capniidae, Leuctridae, Nemouridae, Notonemouridae, and Taeniopterygidae. The phylogenetic relationships within the infraorder Euholognatha were proposed as Scopuridae + (Taeniopterygidae + ((Capniidae + Leuctridae) + (Nemouridae + Notonemouridae))) [1].

Although the family-level relationship within Euholognatha is supported by morphological data, this has never been well supported by molecular evidence. Thomas et al. presented a phylogeny of Plecoptera based on a single gene [10], and the result conflict on some relationships. For instance, the monophyly of Euholognatha and Nemouroidea was not recovered. The family Scopuridae was sister to the suborder Antarctoperlaria, and the superfamily Nemouroidea was separated from the remainder of the Plecoptera [10]. Based on six molecular markers, Terry and Whiting reconstructed phylogenetic relationships among stoneflies [11]. The result demonstrated the monophyly of the Euholognatha, but the family-level relationships are still controversial. The family relationships were recovered as Leuctridae + (Notonemouridae + ((Nemouridae + Capniidae) + (Taeniopterygidae + Scopuridae))) [11]. Recently, South et al. used transcriptomic data to study the phylogeny of North American Plecoptera [12]. They recovered a monophyletic Nemouroidea, but inconsistent tree topologies have been generated by using different methods and datasets. However, the family-level phylogenetic relationships remain unresolved. In addition, mitochondrial genomic data has also been used for the phylogeny of Plecoptera. Nevertheless, more conflicting hypotheses have been proposed. Such as, some studies have proposed controversial phylogenetic relationships about the relative position of Scopuridae and Leuctridae [13–17].

The mitochondrial genome (mitogenome), as an important molecular marker, has been widely used in phylogenetic analyses of various insect orders. However, many factors can affect the phylogenetic reconstruction with mitogenome sequences, such as high A + T content, compositional heterogeneity, and accelerated sequence evolution [18–20]. To evaluate the possible impact of these factors and to reduce artifacts associated with tree reconstruction, an effective method that might be used is to sample more taxa [21–23]. However, many previous studies had limited taxon sampling with only one species per family, causing unstable and inconsistent phylogenetic relationships among euholognathan families [13–17].

To date, approximately thirty mitogenomes of Euholognatha are available in the NCBI database. In this study, we sequenced seven mitogenomes, representing Capniidae, Leuctridae, Taeniopterygidae, and Scopuridae (Table 1). We analyzed the general features and sequence heterogeneity of Euholognatha mitogenomes, and investigated the phylogenetic relationships within Euholognatha. In addition, we used four-cluster likelihood mapping (FcLM) to assess the incongruent relationships among five Nemouroidea families generated by our analyses and previous studies. This study aims to improve our understanding of the phylogeny of these groups.

**Table 1:** Taxonomic information of mitochondrial genomes used in the study

Family	Species	Length	GenBank ID
Capniidae	*Apteroperla tikumana*	15,564	NC_027698
	*Capnia zijinshana*	16,310	KX094942
	*Capnia yunnana*^a^	16,032	ON209193
	*Mesocapnia arizonensis*	14,921	KP642637*
	*Mesocapnia daxingana*	15,524	KY568983*
Leuctridae	*Rhopalopsole bulbifera*	15,599	MK111419*
	*Rhopalopsole subnigra*^a^	15,562	OQ612622*
	*Paraleuctra cercia*	15,625	MK492251
	*Perlomyia kappa*^a^	15,759	OQ612623
	*Perlomyia levanidovae*^a^	15,774	OQ612624
	*Perlomyia isobeae*	15,795	MK492252
Nemouridae	*Nemoura meniscata*	15,895	MN944386
	*Nemoura nankinensis*	16,602	KY940360
	*Nemoura papilla*	15,774	MK290826
	*Amphinemura longispina*	15,709	MH085446
	*Amphinemura bulla*	15,827	MW339348
	*Amphinemura claviloba*	15,707	MN720741
	*Indonemoura jacobsoni*	15,642	MH085448
	*Indonemoura nohirae*	15,738	MH085449
	*Mesonemoura metafiligera*	15,739	MH085450
	*Mesonemoura tritaenia*	15,778	MH085451
	*Protonemura kohnoae*	15,707	MH085452
	*Protonemura orbiculata*	15,758	MH085453
	*Protonemura datongensis*	15,756	MT276842
	*Sphaeronemoura elephas*	15,846	MN944385
	*Sphaeronemoura grandicauda*	15,661	MH085454
	*Sphaeronemoura acutispina*	15,016	MH085455
	*Sphaeronemoura hainana*	15,260	MK111420*
Notonemouridae	*Neonemura barrosi*	14,852	MK111418*
Taeniopterygidae	*Taeniopteryx ugola*	15,353	MG589786
	*Doddsia occidentalis*	16,020	MG589787
	*Taeniopteryx auberti*^a^	15,338	OQ612626
	*Rhabdiopteryx christinae*^a^	15,632	OQ612625
Scopuridae	*Scopura longa*	15,798	MH510071
	*Scopura montana*^a^	15,966	OQ612621
Perlidae (Outgroup)	*Caroperla siveci*	15,353	MG677942
	*Etrocorema hochii*	15,854	MK905888

*Incomplete mitogenome sequence

^a^Mitogenomes sequenced in the present study

## Methods

### Sample collecting, DNA extraction and sequencing

A total of seven species were used in this study, and the collection information was listed in Table S1. All the samples were identified by Weihai Li and Dávid Murányi, and were preserved in 100% ethanol. Total genomic DNA for each specimen was extracted using DNeasy Blood & Tissue Kit (Qiagen, Germany) according to the manufacturer’s instructions. The voucher specimens and extracted DNA were stored at − 20℃ until used.

Genomic DNA with qualified concentration was submitted to Berry Genomics Co., Ltd. (Beijing, China) for library construction and high-throughput sequencing. An Illumina TruSeq library with average insert sizes of approximately 350 bp was generated and then sequenced as 150 bp paired-end runs on the Illumina HiSeq 2500 platform.

### Sequence assembly, annotation and analyses

The mitogenome assembly strategy refers to our previous studies [13, 16, 24–27]. Each library generated about 10 Gb of raw data. Then, raw reads were filtered using Trimmomatic v0.30 with default parameters [28]. Clean data were subject to de novo assembling using IDBA-UD [29] with the parameters: similarity threshold 98%, minimum k value 80, and maximum k value 240. All the newly sequenced mitogenomes have already been deposited in GenBank, and the detailed information is listed in Table S1.

All 22 transfer RNA genes (tRNAs) were annotated using MITOS web server [30]. Protein-coding genes (PCGs) and ribosomal RNA genes (rRNAs) were identified by alignment with their homologous genes.

Nucleotide composition was analyzed with MEGA 7.0 [31]. Composition skew values were obtained with AT skew = (A − T)/(A + T) and GC skew = (G − C)/(G + C) [32]. The rates of non-synonymous substitutions (Ka) and the rate of synonymous substitutions (Ks) for PCGs were determined with DnaSP 5.0 [33].

### Phylogenetic analyses

For phylogenetic reconstruction, the 13 PCGs and two rRNAs of the 7 samples sequenced here, plus 28 published euholognathan mitogenomes and two perlid mitogenomes (used as outgroups) were used (Table 1). Each PCG was aligned with MAFFT algorithm as implemented in TranslatorX online using codon–based multiple alignments [34]. Two rRNAs were aligned individually using the G-INS-I strategy in MAFFT online [35], and ambiguously aligned regions masked with Gblocks [36]. All alignments were imported into MEGA 7.0 and concatenated into four datasets: (1) PCG matrix, including all codon positions of PCGs with 11,181 nucleotides; (2) PCGR matrix, including 13 PCGs plus two rRNA genes with 13,191 nucleotides; (3) PCG12 matrix, including the first and second codon positions of 13 PCGs with 7,454 nucleotides; (4) PCG12R matrix, including the first and second codon positions of 13 PCGs plus two rRNAs with 9,464 nucleotides. The heterogeneity of sequence divergence within the two datasets was analyzed using AliGROOVE [37] with default parameters. To evaluate single phylogenetic splits, FcLM analysis was conducted using TreePuzzle v5.3 [38].

ModelFinder was used to select the best–fit partitioning schemes for each dataset [39]. According to the Akaike Information Criterion (AIC), the best schemes were selected and subsequently employed in Bayesian inference (BI) and maximum likelihood (ML) analyses (Table S2). IQ–TREE [39] and MrBayes 3.2.6 [40] were used to construct the ML and BI tree, respectively. For ML analysis, phylogenetic trees were generated using an ultrafast bootstrap approximation with 1000 replicates. For MrBayes, parameters were set as follows: two simultaneous chains running for 10 million generations, sampling a tree every 1000 generations, and discarding the first 25% as burn-in.

## Results

### General features of Euholognathan mitogenomes

For the comparative analyses, 29 of the 35 species have complete mitogenomes (Table 1). All the euholognathan mitogenomes have 37 typical genes (i.e., 22 tRNAs, 13 PCGs, and two rRNAs) and a control region, as has been reported in other published stoneflies and insects [13–18, 20]. Gene order was consistent with the ancestral gene order of *Drosophila yakuba* [41].

**Fig. 1:**
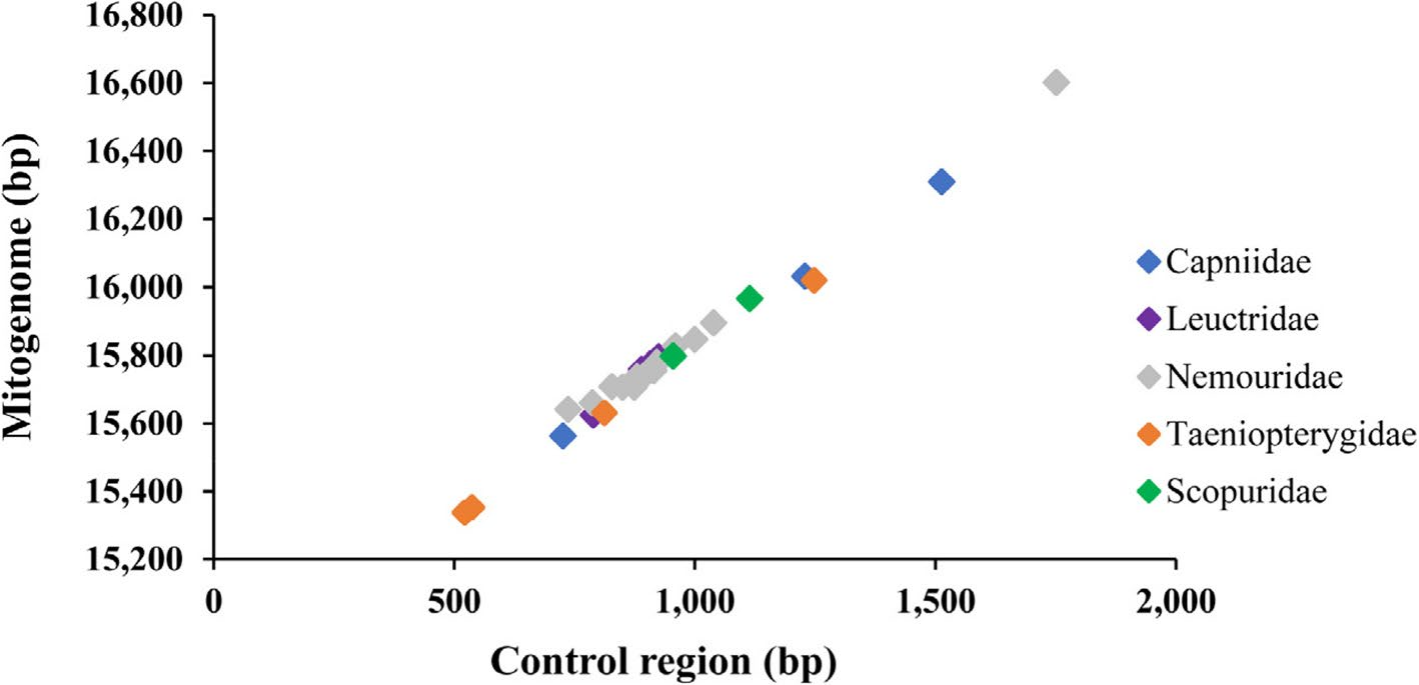
Size of the complete mitogenomes and complete control regions of 29 euholognathan species

The complete mitogenomes of the 29 euholognathan species ranged from 15,016 bp (*Sphaeronemoura acutispina*) to 16,602 bp (*Nemoura nankinensis*) in length (Fig. 1; Table 1). The length variation in control regions is mostly responsible for the observed length variation among mitogenomes (Fig. 1). All 35 mitogenomes showed a strong AT bias with an average A + T content of 69.1% ranging from 66.3% (*Amphinemura longispina*) to 71.9% (*Perlomyia kappa*) (Table S3). Furthermore, all 35 mitogenomes presented a positive AT–skew (from 0.01 in *Capnia zijinshana* to 0.08 in *A. longispina*) and a negative GC–skew (from − 0.15 in *Doddsia occidentalis* to − 0.28 in *Scopura montana*) for the whole mitogenome, which is typical in insect mitogenomes [13–18].

### Sequence heterogeneity in Euholognathan mitogenomes

**Fig. 2:**
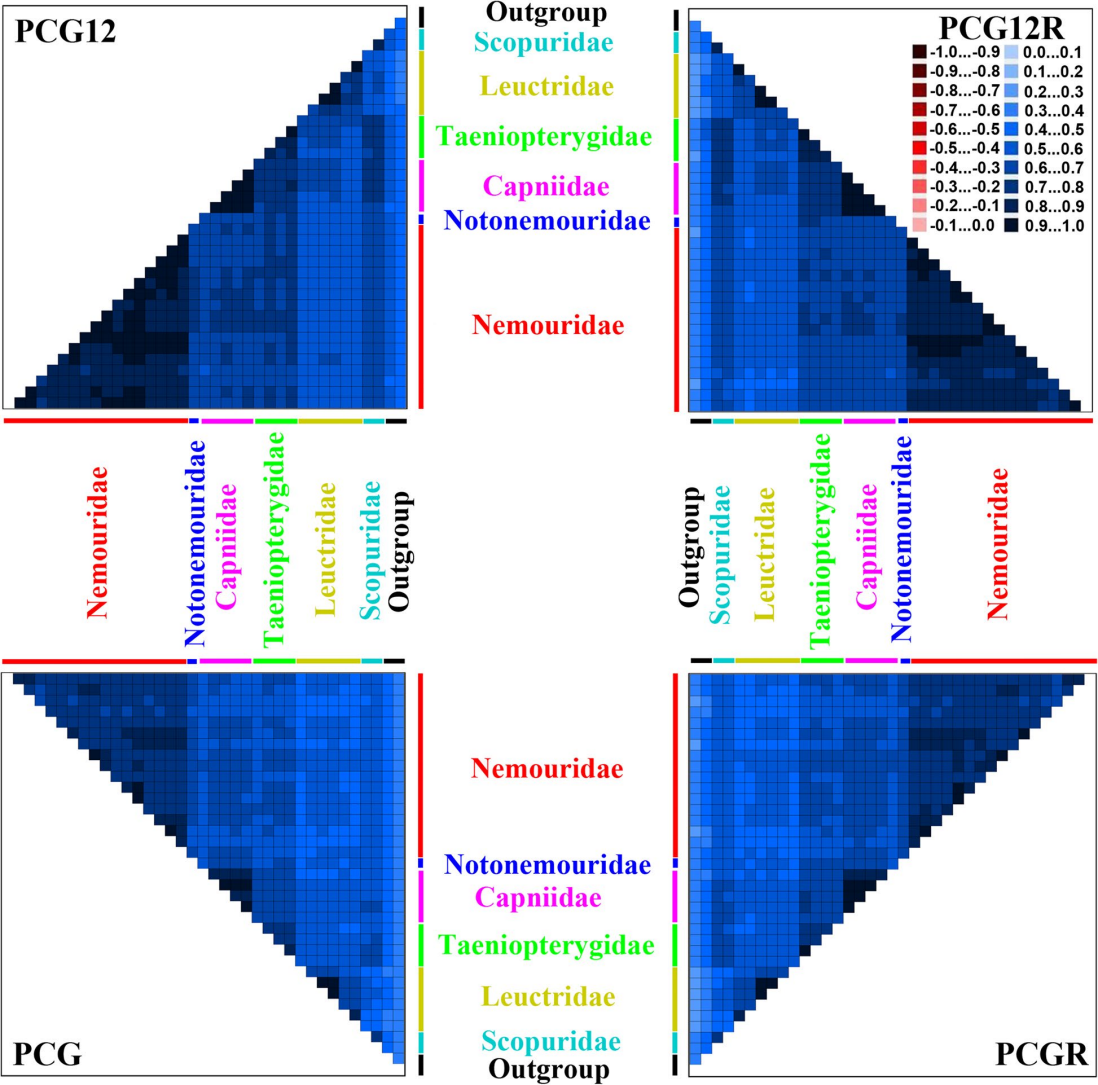
Heterogeneous sequence divergence within Euholognatha mitogenomes. The mean similarity score between sequences is represented by a colored square, based on AliGROOVE scores ranging from − 1, indicating the great difference in rates from the remainder of the data set, that is, heterogeneity (red coloring), to + 1, indicating rates match all other comparisons (blue coloring)

The sequence heterogeneity analysis found low heterogeneity in sequence divergence for a subset of taxa (Fig. 2). The degree of heterogeneity of the PCG12 (mean similarity score, 0.654) and PCG12R datasets (0.644) were lower than that of the PCG (0.574) and PCGR datasets (0.568) (Fig. 2), suggesting that third codon positions are more heterogeneous than other two codon positions. This finding was supported by additional research on sequence divergence in datasets defined by codon position alone (Fig. S1). Because negative similarity scores were found in most pairwise sequence comparisons of the third codon position (Fig. S1). In addition, sequence heterogeneity for leuctrid species displayed relatively lower similarity scores (Fig. 2), suggesting that the leuctrid mitogenomes are more heterogeneous than other euholognathan families.

**Fig. 3:**
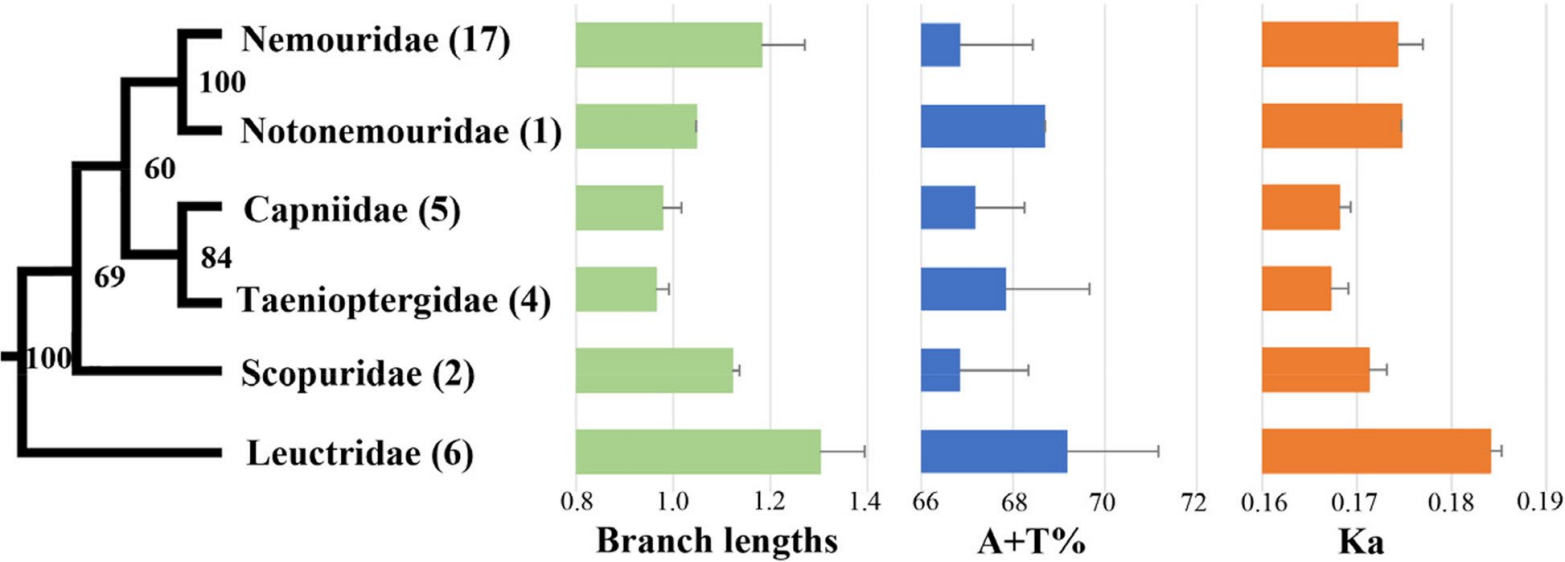
Systematic errors in phylogenetic analyses under site-homogeneous models. The tree was obtained by ML analysis of the PCG dataset. Numbers close to the branching points are bootstrap proportion (BP). The numbers in the brackets indicate the number of species used for phylogenetic analyses in the corresponding taxa. The branch lengths were calculated by IQ–TREE based on PCG dataset. The A + T content (%), and rate of non–synonymous substitutions (Ka) were calculated from the protein–coding genes. Error bars represent standard deviations from the data of multiple species

We investigated the compositional diversity of nucleotides of mitochondrial PCGs across the available euholognathan mitogenomes (Fig. 3). The A + T content in Leuctridae (69.18 ± 1.98%) was higher than that in other families, but the degree of heterogeneity among euholognathan mitogenomes at the family level was low (from 66.85 ± 1.48% to 69.18 ± 1.98%). In addition, *Ka* was low for all euholognathan mitogenomes (< 0.200). However, the average *Ka* in Leuctridae (0.184 ± 0.001) was significantly higher than that in other families, suggesting an accelerated evolutionary rate in Leuctridae.

### Phylogenetic and four–cluster likelihood mapping (FcLM) analyses

**Fig. 4:**
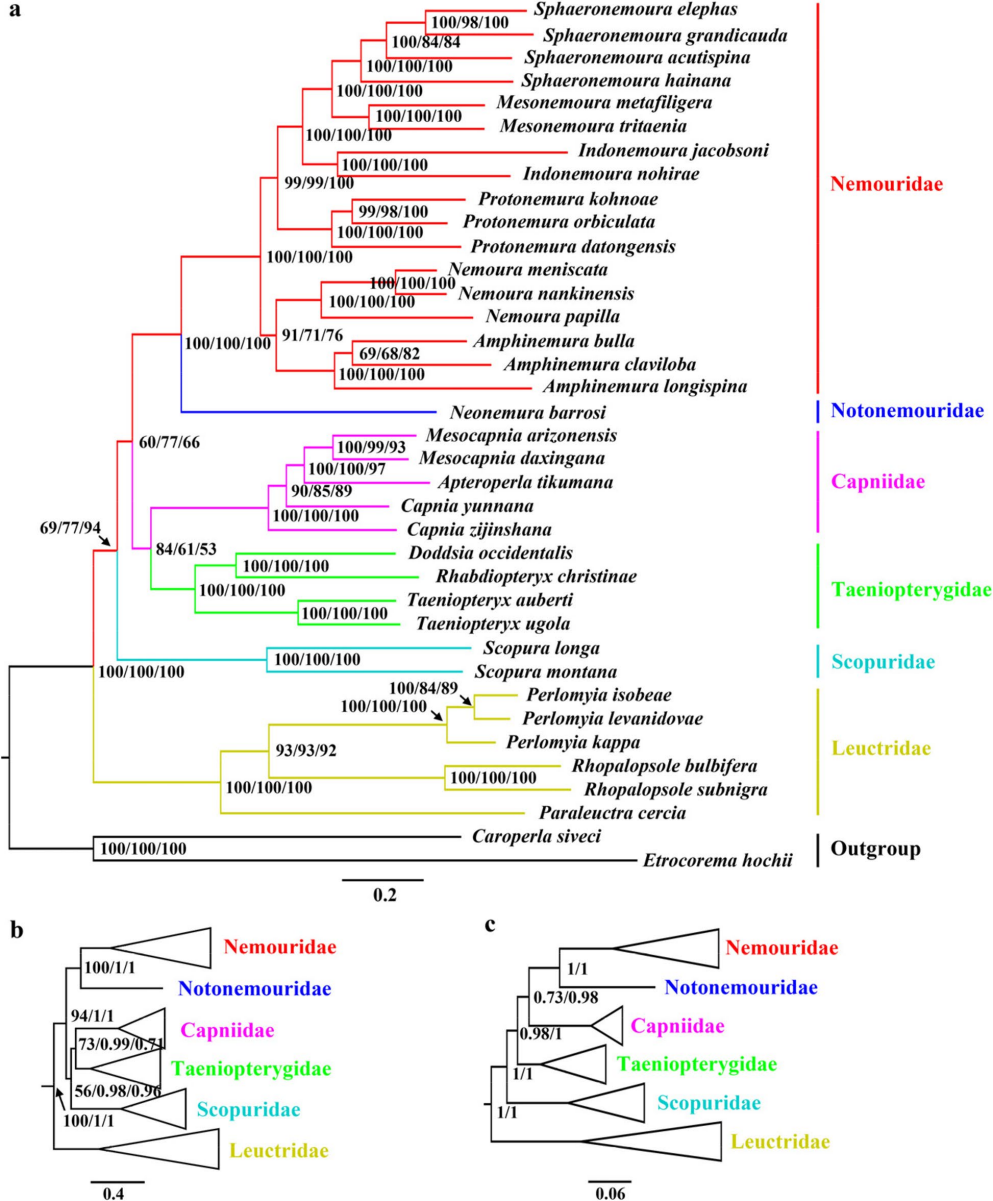
Phylogenetic trees obtained from the Bayesian inferences and maximum–likelihood analyses. **a **The congruent topology from the analysis of ML–PCG (BP in left), ML–PCG12 (BP in middle), and ML–PCG12R (BP in right); **b** The congruent topology from the analyses of ML–PCGR (BP in left), BI–PCG (PP in middle), and BI–PCGR (PP in right); **c** The congruent topology from the analyses of BI–PCG12 (PP in left), BI–PCG12R (PP in right). Values at node represented the Bayesian posterior probabilities (PP) or bootstrap probabilities (BP). We showed a schematic version of the trees (**b**–**c**) with some ingroups collapsed and outgroups removed for clarity

In this study, BI and ML trees were inferred for each of the four datasets (Table S2). Although ML and BI analysis showed inconsistent topologies across the different datasets, some relationships were highly supported in most of the analyses (Fig. 4). Such as, Nemouridae was sister to Notonemouridae in all analyses with strong support (Bayesian posterior probabilities (PP) = 1.00, bootstrap probabilities (BP) = 100), and Leuctridae was recovered at the basal position of the tree (PP = 1.00, BP = 100). The sister group of Capniidae and Taeniopterygidae was supported by most BI and ML analyses, but most support values were relatively low (PP = 0.71/0.99, 53 ≤ BP ≤ 84) (Fig. 4a and b). In these topologies, Scopuridae was the sister group to all the rest of the Nemouroidea families (Fig. 4a and c) or to the clade Capniidae plus Taeniopterygidae (Fig. 4b). Our result shows that regardless of the position of Scopuridae, Nemouroidea would be recovered as non-monophyletic, as the positioning of Leuctridae remained unchanged (Fig. 4).

**Fig. 5:**
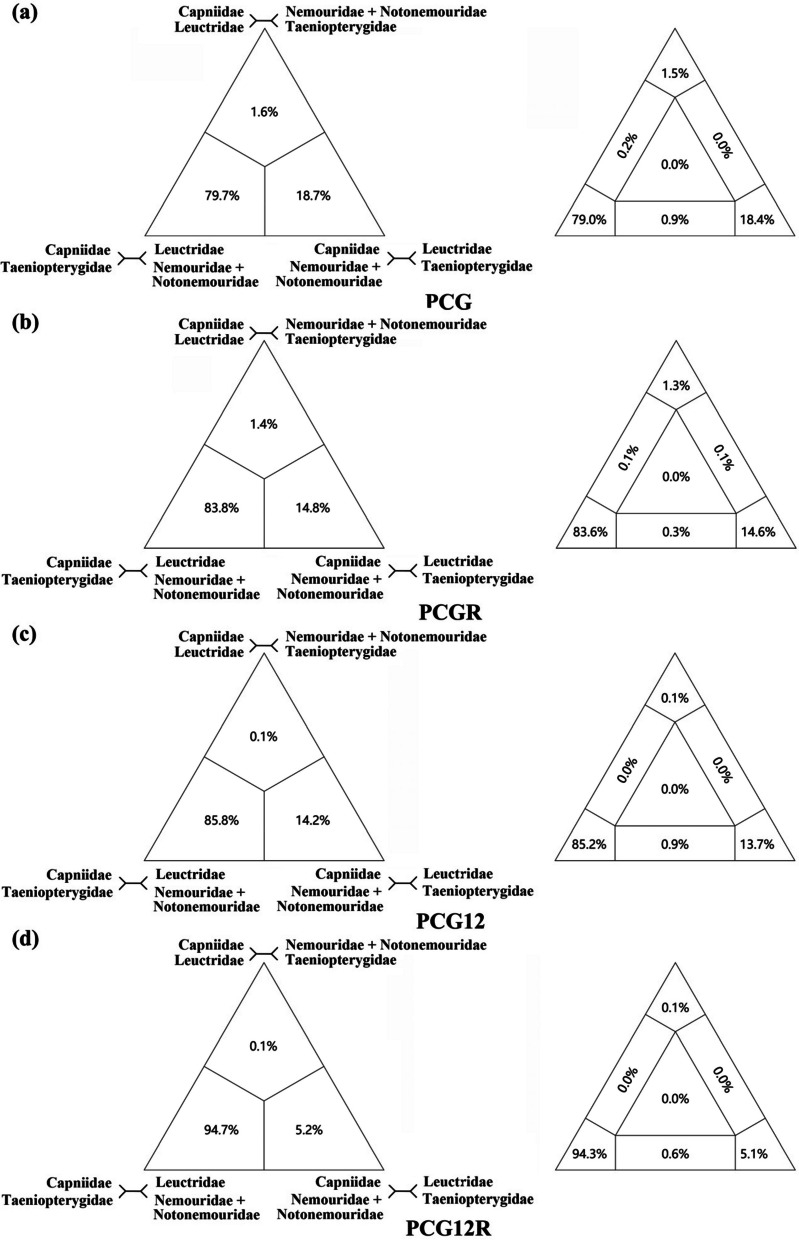
Results of Four-cluster Likelihood Mapping as 2D simplex graphs. **a** Four-cluster Likelihood Mapping based on PCG; **b** four-cluster Likelihood Mapping based on PCGR; **c** four-cluster Likelihood Mapping based on PCG12; **d** four-cluster Likelihood Mapping based on PCG12R

To evaluate the incongruent relationships among the five Nemouroidea families generated by our analysis and previous studies, particularly whether Capniidae is grouped with Taeniopterygidae or Leuctridae, we excluded the family Scopuridae and conducted four-cluster likelihood mapping (FcLM) analyses. The FcLM analysis preferred for the sister group relationship of Capniidae and Taeniopterygidae (79.7%/83.8%/85.8%/94.7%) (Fig. 5). Alternative relationships were weakly supported: Capniidae as the sister group to Leuctridae (1.6%/1.4%/0.1%/0.1%), and Capniidae as sister group to Nemouridae plus Notonemouridae (18.7%/14.8%/14.2%/5.2%). Moreover, considering the unstable phylogenetic position of Scopuridae and the potential noise introduced by certain species in the analysis, we also excluded the family Scopuridae and reconstructed the phylogenetic relationships among the five Nemouroidea families. Across various datasets and analysis methods, consistent tree topologies were obtained (Fig. S2). The results supported the basal position of Leuctridae (all PPs = 1, BPs = 100) and the sister group relationship between Capniidae and Taeniopterygidae (all PPs = 1, 85 ≤ BP ≤ 94).

## Discussion

In this research, seven mitogenomes from the infraorder Euholognatha were sequenced. We found a low heterogeneity among the euholognathan families, and a relatively high compositional heterogeneity in Leuctridae. Therefore, leuctrid species may be placed in an unstable or possibly misplaced position on phylogenetic trees, as previous studies have shown that the compositional variability of mitogenomes in particular groups may lead to the improper grouping of unrelated taxa [18–20].

Recently, many studies have reported the highly A + T content, accelerated substitution rates, and relatively high compositional heterogeneity in some groups of insect mitogenomes [20, 42–44]. These potential biases are major sources of systematic error in phylogenetic reconstruction, leading to topological contradiction with morphological and/or other molecular datasets [20, 23, 43]. Previous studies found a low degree of sequence heterogeneity within Systellognatha and Plecoptera [14, 27]. In this study, analyses of sequence divergence, base composition, and substitution rates also revealed a low degree of compositional heterogeneity in euholognathan mitogenomes. Meanwhile, leuctrid mitogenomes have the highly A + T content, accelerated substitution rates, and relatively high compositional heterogeneity, indicating low nodal support values and unstable phylogenetic relationships among the corresponding families may occur in phylogenetic reconstructions.

In the current study we reconstructed the family-level phylogenetic relationships within Euholognatha. The monophyly of Euholognatha is evidenced by three morphological characteristics: a single corpus allatum, a soft egg chorion, and the crossing of segmental nerves under longitudinal abdominal muscles [1]. Drumming and related female structures can easily distinguish Scopuridae from Nemouroidea, and five Nemouroidea families can be distinguished by the sperm transfer mode and related morphological changes [1, 45]. The monophyly of each euholognathan family was well supported by morphological data, and the Scopuridae are the sister group of the large superfamily Nemouroidea [1]. However, the relationships among the Nemouroidea families were controversial for a long time. An interesting arrangement occurred in Nemouroidea, because the northern hemisphere distributed nemourids and the southern hemisphere distributed notonemourids were listed as sister groups by morphological studies [1, 8, 46]. This arrangement differs from the previous morphological hypothesis (Illies place Leuctridae as the sister of Nemouroidae [6]) and some molecular analyses [10, 11]. In addition, the basal position of Nemouroidea is also controversial. Previous morphological studies placed Taeniopterygidae at the base of Nemouroidea [1], while Leuctridae was supported as the earliest branching group by most molecular analyses [11, 13, 24, 25]. Although our analyses generated three inconsistent topologies, the basal position of Leuctridae was recovered by all analyses (Fig. 4). Comparable to many previous phylogenetic studies, the sister groups of Nemouridae plus Notonemouridae and Capniidae plus Taeniopterygidae were also recovered by most mitogenome studies [13, 16, 17, 24, 25, 47]. Although morphological analyses have supported the sister group of Nemouridae and Notonemouridae, the clade Capniidae plus Taeniopterygidae and the position of Leuctridae remain in conflict with the morphological findings [1].

In contrast to morphological studies, there is no doubt about the relationship between Nemouridae and Notonemouridae, and the debate has mainly focused on whether Taeniopterygidae or Leuctridae is the sister group of Capniidae. After excluding mitogenomes of the family Scopuridae, the FcLM analysis tends to support a sister group relationship between Capniidae and Taeniopterygidae (Fig. 5). The sister group of Capniidae and Taeniopterygidae is also supported by the phylogenetic analysis results after excluding two scopurid mitogenomes (Fig. S2). These results were consistent with most tree topologies based on four datasets (Fig. 4a and b) and most mitogenome studies [13, 16, 17, 24, 25, 47], but inconsistent with Zwick’s phylogenetic analyses [1].

**Fig. 6:**
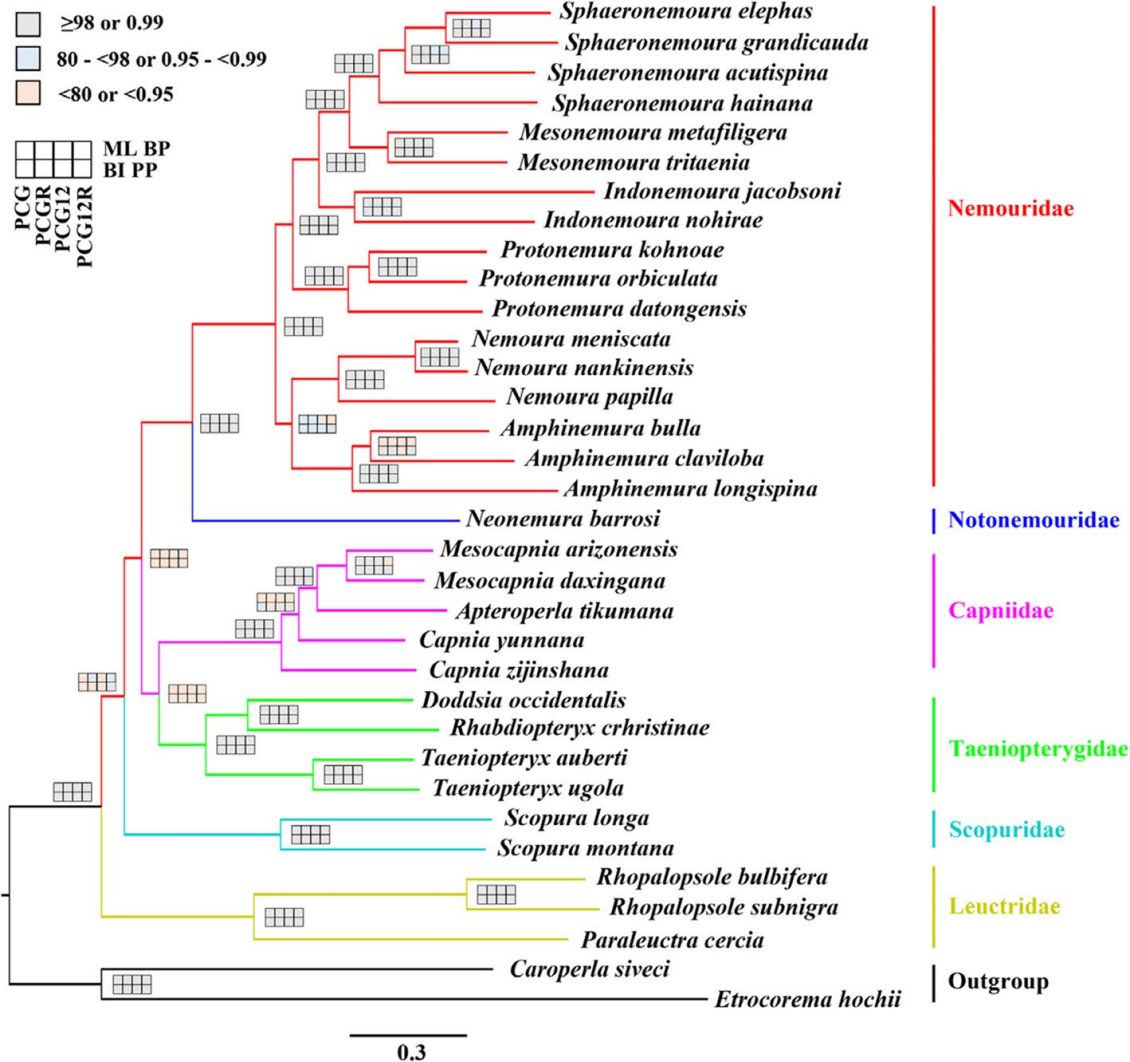
Molecular phylogeny of Euholognatha. Topology refers to the ML tree generated in IQ-TREE based on PCG dataset. Nodal supports from various analyses of different datasets are shown as squares at the nodes, with explanation of different colours shown in the bottom left box. PP and BP mean Bayesian posterior probabilities and bootstrap probabilities, respectively

According to the result of sequence heterogeneity, leuctrid mitogenomes are more heterogeneous than other euholognathan families, which may affect the relationships among corresponding families in phylogenetic reconstruction. Here, we used the same methods to reconstruct an additional eight phylogenetic trees with a reduced number of leuctrid taxa to see if certain species within Leuctridae had an obvious impact on topologies. After removing three leuctrid species with relatively high compositional heterogeneity,, our results provide more stable phylogenetic relationships (Fig. 6). All BI and ML trees had the same topological structures. The monophyly of Nemouroidea was not supported, and the relationships among six Euholognatha families were recovered as: Leuctridae + (Scopuridae + ((Taeniopterygidae + Capniidae) + (Nemouridae + Notonemouridae))). This result is consistent with the findings in Fig. 4a, indicating that reducing certain leuctrid species contributes to obtaining a more consistent topology.

Our results provide a new insight into the phylogeny of Euholognatha. Although the euholognathan mitogenomes showed a low degree of compositional heterogeneity, a more heterogeneous Leuctridae can indeed affect the phylogenetic reconstruction. In addition, using mitogenome data alone may not be sufficient to recover the relationships among euholognathan families, as evidenced by the relatively low support values of some family-level relationships. Furthermore, mitogenomes of these euholognathan families (especially Scopuridae and Notonemouridae) are still limited, and errors may be introduced in the phylogenetic reconstruction of these clades. Dense sampling of mitogenomes can serve as an effective approach to enhance estimations of molecular rates and variations in base composition, thereby producing robustly supported phylogenetic inferences [21, 23]. Therefore, combining other types of data (such as morphological characters and nuclear genes) and adding taxonomic samples are needed to resolve these problems.

## Conclusions

In this research, seven mitogenomes from the infraorder Euholognatha were sequenced. We found a low heterogeneity among the euholognathan families, and a relatively high compositional heterogeneity in Leuctridae. Our analysis generated different tree topologies, and the position of some families was different from the currently accepted phylogeny. These conflicting topologies may result from employing different strategies for taxon sampling, utilizing diverse types of data, and applying various phylogenetic methods (e.g., substitution models). Nonetheless, the conflicting topologies reflect the complex phylogenetic signals present in the sequence data, particularly in the mitogenome sequences of Euholognatha. Our study found that by separately excluding sparsely sampled Scopuridae or high heterogeneity leuctrid taxa, the impact of these data on phylogenetic reconstruction can be effectively reduced, resulting in a more consistent tree topology. Although these results have not yet reconstructed the monophyly of Nemouroidea and differ somewhat from morphological results, like most molecular studies, they all support the sister group relationship between Taeniopterygidae and Capniidae. Finally, based on the results of this study, we reconstructed the relationships within Euholognatha as: Leuctridae + (Scopuridae + ((Taeniopterygidae + Capniidae) + (Nemouridae + Notonemouridae))). However, considering the relatively low support values for certain nodes, the position of some families (especially Scopuridae) still requires further investigation.

### Supplementary Information

**Additional file 1:** **Fig. S1. **AliGROOVE analyses of the codon position of protein-coding genes. **Fig. S2. **Molecular phylogeny of Nemouroidea. **Table S1. **Information of Euholognatha species newly sequenced in the present study. **Table S2**. Best partitioning scheme and model selected by ModelFinder for phylogenetic analyses. **Table S3.** Mitochondrial nucleotide composition in 35 Euholognathan stoneflies.

## Data Availability

All the accession numbers and the newly sequenced (ON209193, OQ612621, OQ612622, OQ612623, OQ612624, OQ612625, OQ612626) and assembled mitogenomes for seven accessions of euholognathan species have been uploaded to the National Center for Biotechnology Information database.
