# Supplementary material for: Mitochondrial genomes provide insights into the Euholognatha (Insecta: Plecoptera)
Source: BMC Ecol Evol. 2024 Feb 1;24:16. doi: 10.1186/s12862-024-02205-6 (PMC10832105; doi:10.1186/s12862-024-02205-6)
Supplement: Supplementary file 1 — Additional file 1: Fig. S1. AliGROOVE analyses of the codon position of protein-coding genes. Fig. S2. Molecular phylogeny of Nemouroidea. Table S1. Information of Euholognatha species newly sequenced in the present study. Table S2. Best partitioning scheme and model selected by ModelFinder for phylogenetic analyses. Table S3. Mitochondrial nucleotide composition in 35 Euholognathan stoneflies. [file 12862_2024_2205_MOESM1_ESM.docx]

**Supplementary figures and tables**


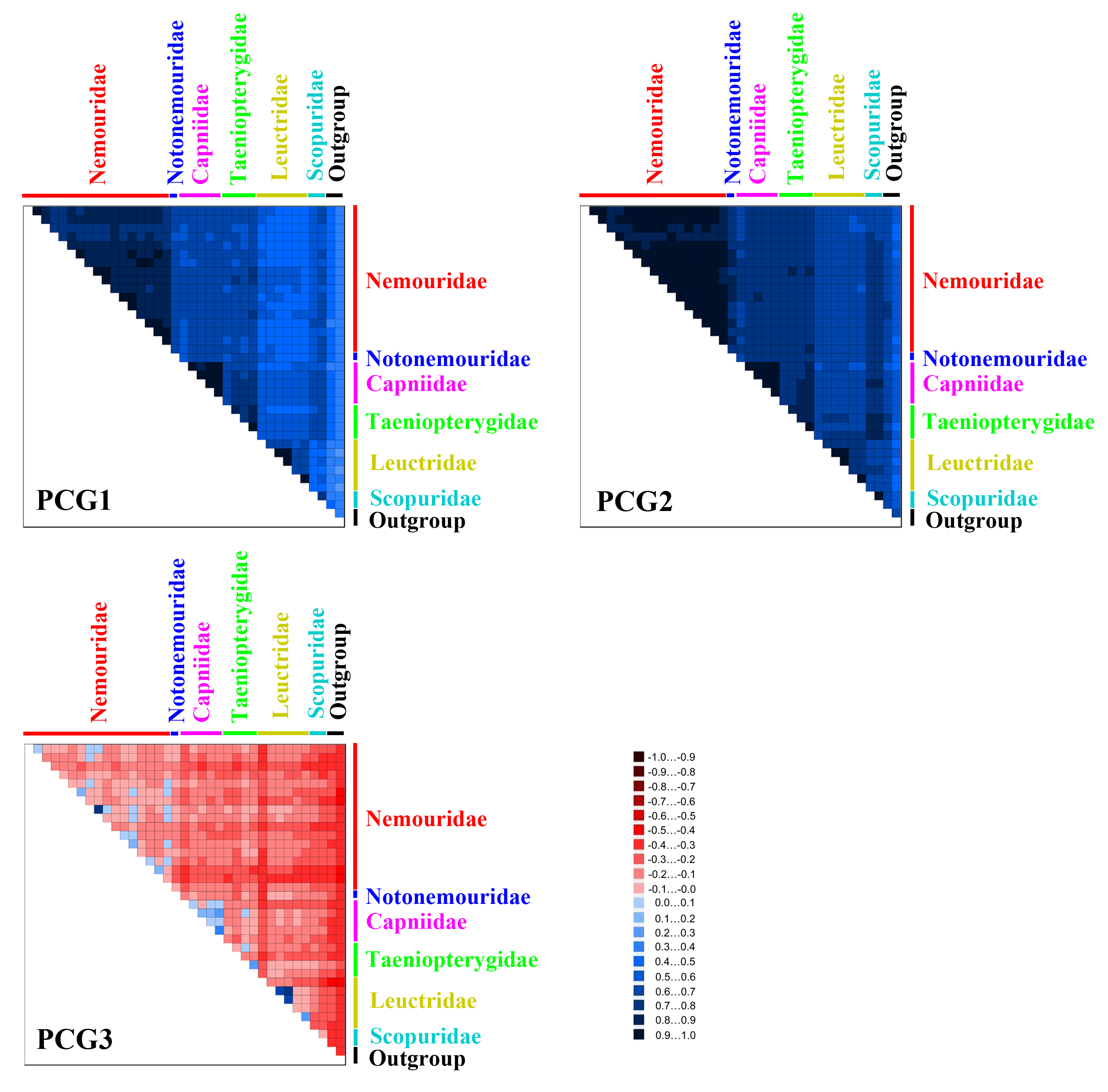


**Fig. S1.** AliGROOVE analyses of the codon position of protein-coding genes. The obtained mean similarity score between sequences was represented by a colored square. The scores were ranging from -1, indicating full random similarity, to +1, non-random similarity. The darker red indicated the higher randomized accordancy between pairwise sequence comparisons. Blue indicated the opposite.

**
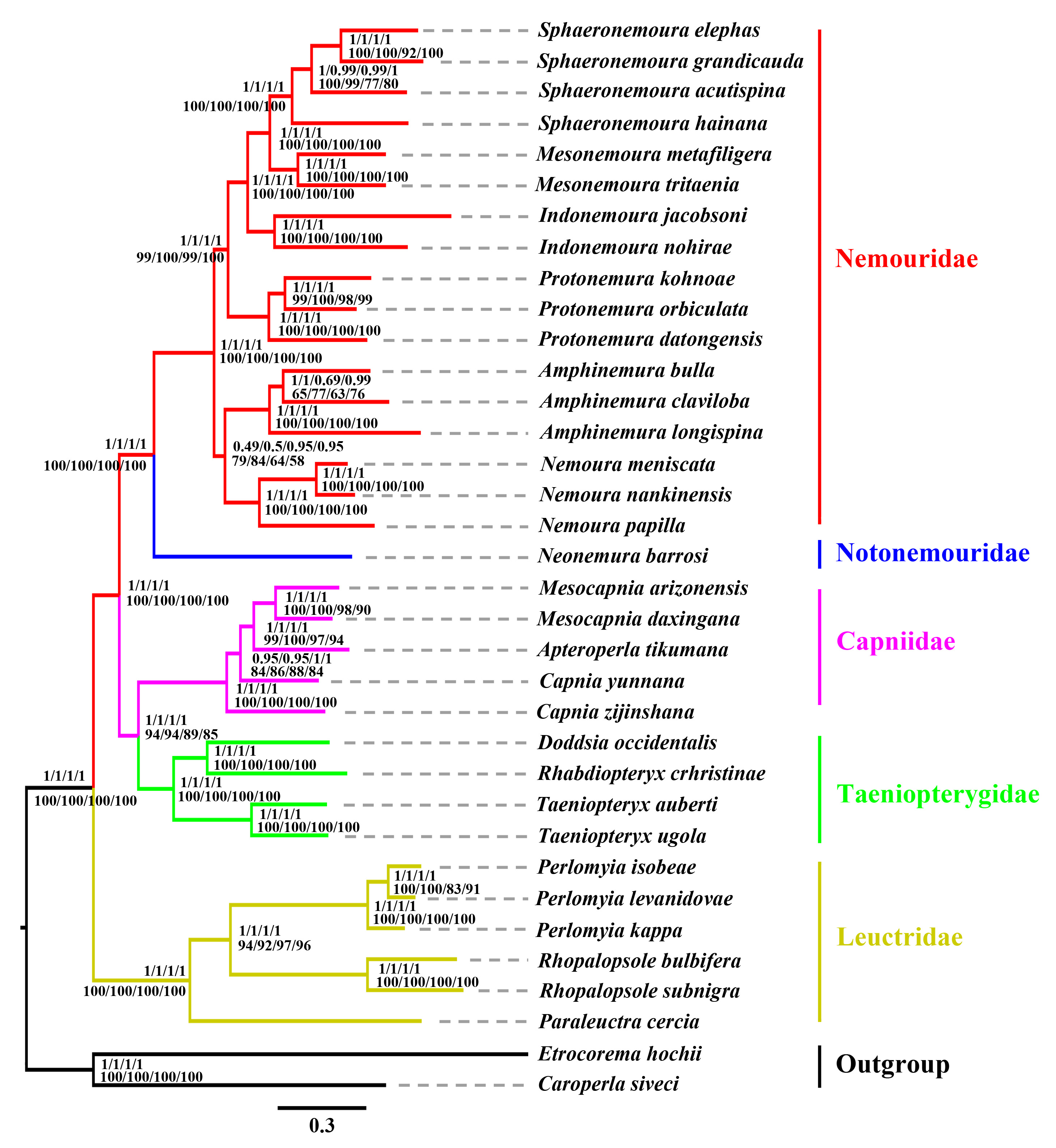
**

**Fig. S2.** Molecular phylogeny of Nemouroidea. Topology refers to the ML tree generated in IQ-TREE based on PCG dataset. Values at nodes are Bayesian posterior probabilities (PP) and ML bootstrap values (BP) using the PCG/PCGR/PCG12/PCG12R (up) and PCG/PCGR/PCG12/PCG12R (down) datasets.

**Table S1.** Information of Euholognatha species newly sequenced in the present study

| Species | Locality | Time | Museum No. |
| --- | --- | --- | --- |
| *Capnia yunnana* | Sejila Mts, Tibet, China | 5-5-2017 | VHem-0033 |
| *Rhopalopsole subnigra* | Kan'onji city, Kagawa pref, Japan | 6-17-2016 | VHem-0090 |
| *Perlomyia kappa* | Saijo city, Ehime pref, Japan | 5-22-2016 | VHem-0094 |
| *Perlomyia levanidovae* | Chiak Mts, Gangwon province, South Korea | 5-14-2016 | VHem-0095 |
| *Taeniopteryx auberti* | Poľana Mts, Banskobystrickỷregion, Slovakia | 6-4-2018 | VHem-0227 |
| *Rhabdiopteryx christinae* | Sierra de Huetor Natural Park, Granada, Southern Spain | 6-15-2018 | VHem-0211 |
| *Scopura montana* | Chojamine Mts, Nagano pref., Japan | 11-3-2015 | VHem-0014 |

* Incomplete genome sequence.

**Table S2**. Best partitioning scheme and model selected by ModelFinder for phylogenetic analyses.

| Dataset and Partition type | Subset Partitions and Model for MrBayes | Subset Partitions and Model for IQ-TREE |
| --- | --- | --- |
| PCG gene partition | P1: (ATP6, CYTB): GTR + I + G  P2: (ATP8): GTR + I + G  P3: (COI): GTR + I + G  P4: (COII, COIII, ND3): GTR + I + G  P5: (ND1, ND4, ND4L, ND5): GTR + I + G  P6: (ND2, ND6): GTR + I + G | P1: (ATP6): TIM2 + I + G  P2: (ATP8): TPM3u + I + G  P3: (COI): GTR + I + G  P4: (COII): TIM2 + I + G  P5: (COIII): TIM2 + I + G  P6: (CYTB): TIM2 + I + G  P7: (ND1): TIM + I + G  P8: (ND2): TIM3 + I + G  P9: (ND3): TIM2 + I + G  P10: (ND4): TVM + I + G  P11: (ND4L): TVM + I + G  P12: (ND5): TVM + I + G  P13: (ND6): TIM3 + I + G |
| PCGR gene partition | P1: (ATP6): GTR + I + G  P2: (ATP8, ND3): GTR + I + G  P3: (COI): GTR + I + G  P4: (COII, COIII): GTR + I + G  P5: (ND1, ND4, ND4L, ND5): GTR + I + G  P6: (ND2, ND6): GTR + I + G  P7: (12srRNA, 16srRNA): GTR + I + G | P1: (ATP6): TIM2 + I + G  P2: (ATP8): TPM3u + I + G  P3: (COI): TIM2 + I + G  P4: (COII): TIM2 + I + G  P5: (COIII): TIM2 + I + G  P6: (CYTB): TIM2 + I + G  P7: (ND1): GTR + I + G  P8: (ND2): TIM3 + I + G  P9: (ND3): TIM2 + I + G  P10: (ND4): TVM + I + G  P11: (ND4L): TVM + I + G  P12: (ND5): GTR + I + G  P13: (ND6): TIM3 + I + G  P14: (12srRNA): GTR + I + G  P15: (16srRNA): GTR + I + G |
| PCG (excluding Leuctridae) gene partition | P1: (ATP6, CYTB): GTR + I + G  P2: (ATP8): GTR + I + G  P3: (COI): GTR + I + G  P4: (COII, COIII, ND3): GTR + I + G  P5: (ND1, ND4, ND4L, ND5): GTR + I + G  P6: (ND2, ND6): GTR + I + G | P1: (ATP6): TIM2 + I + G  P2: (ATP8): TPM3u + I + G  P3: (COI): GTR + I + G  P4: (COII): TIM2 + I + G  P5: (COIII): TIM2 + I + G  P6: (CYTB): TIM2 + I + G  P7: (ND1): GTR + I + G  P8: (ND2): TVM + I + G  P9: (ND3): TIM2 + I + G  P10: (ND4): TVM + I + G  P11: (ND4L): TVM + I + G  P12: (ND5): TVM + I + G  P13: (ND6): TIM3 + I + G |
| PCGR (excluding Leuctridae) gene partition | P1: (ATP6): GTR + I + G  P2: (ATP8): GTR + I + G  P3: (COI): GTR + I + G  P4: (COII, COIII, ND3): GTR + I + G  P5: (ND1, ND4, ND4L, ND5): GTR + I + G  P6: (ND2, ND6): GTR + I + G  P7: (12srRNA, 16srRNA): GTR + I + G | P1: (ATP6): TIM2 + I + G  P2: (ATP8): TPM3u + I + G  P3: (COI): TIM2 + I + G  P4: (COII): TIM2 + I + G  P5: (COIII): GTR + I + G  P6: (CYTB): GTR + I + G  P7: (ND1): GTR + I + G  P8: (ND2): GTR + I + G  P9: (ND3): GTR + I + G  P10: (ND4): TVM + I + G  P11: (ND4L): TVM + I + G  P12: (ND5): GTR + I + G  P13: (ND6): GTR + I + G  P14: (12srRNA): GTR + I + G  P15: (16srRNA): GTR + I + G |

**Table S3.** Mitochondrial nucleotide composition in 35 Euholognathan stoneflies.

| Family | Species | Whole mitogenome | | |
| --- | --- | --- | --- | --- |
|  |  | A+T% | AT-skew | GC-skew |
| Capniidae | *Apteroperla tikumana* | 66.5 | 0.03 | –0.19 |
|  | *Capnia zijinshana* | 68.5 | 0.01 | –0.18 |
|  | *Capnia yunnana* | 69.2 | 0.03 | -0.19 |
|  | *Mesocapnia arizonensis* | 68.5 | 0.02 | –0.17 |
|  | *Mesocapnia daxingana* | 68.1 | 0.02 | –0.18 |
| Leuctridae | *Rhopalopsole bulbifera* | 70.7 | 0.02 | –0.20 |
|  | *Rhopalopsole subnigra* | 69.7 | 0.03 | -0.21 |
|  | *Paraleuctra cercia* | 67.4 | 0.04 | –0.21 |
|  | *Perlomyia kappa* | 71.9 | 0.04 | -0.19 |
|  | *Perlomyia levanidovae* | 71.5 | 0.04 | -0.19 |
|  | *Perlomyia isobeae* | 71.8 | 0.03 | –0.19 |
| Nemouridae | *Nemoura meniscata* | 70.8 | 0.04 | –0.19 |
|  | *Nemoura nankinensis* | 71.2 | 0.04 | –0.18 |
|  | *Nemoura papilla* | 68.0 | 0.06 | -0.21 |
|  | *Amphinemura longispina* | 66.3 | 0.08 | -0.26 |
|  | *Amphinemura bulla* | 68.9 | 0.06 | -0.22 |
|  | *Amphinemura claviloba* | 68.5 | 0.07 | -0.23 |
|  | *Indonemoura jacobsoni* | 69.3 | 0.06 | -0.23 |
|  | *Indonemoura nohirae* | 70.9 | 0.04 | -0.21 |
|  | *Mesonemoura metafiligera* | 69.1 | 0.05 | -0.21 |
|  | *Mesonemoura tritaenia* | 68.6 | 0.05 | -0.21 |
|  | *Protonemura kohnoae* | 69.2 | 0.04 | -0.19 |
|  | *Protonemura orbiculata* | 69.7 | 0.04 | -0.19 |
|  | *Protonemura datongensis* | 68.8 | 0.04 | -0.18 |
|  | *Sphaeronemoura elephas* | 67.1 | 0.07 | -0.24 |
|  | *Sphaeronemoura grandicauda* | 66.8 | 0.07 | -0.23 |
|  | *Sphaeronemoura acutispina* | 68.8 | 0.06 | -0.22 |
|  | *Sphaeronemoura hainana* | 69.0 | 0.06 | -0.23 |
| Notonemouridae | *Neonemura barrosi* | 69.8 | 0.06 | -0.22 |
| Taeniopterygidae | *Doddsia occidentalis* | 68.4 | 0.02 | –0.15 |
|  | *Taeniopteryx ugola* | 69.8 | 0.02 | –0.21 |
|  | *Taeniopteryx auberti* | 71.2 | 0.04 | -0.19 |
|  | *Rhabdiopteryx christinae* | 67.4 | 0.03 | -0.20 |
| Scopuridae | *Scopura longa* | 69.2 | 0.02 | -0.26 |
|  | *Scopura montana* | 67.5 | 0.05 | -0.28 |
